# Cod Liver Oil, as a Curative Means, Physiologico-Pathologically Illustrated by a Variety of Facts and Experiments on Animals

**Published:** 1842-10

**Authors:** 


### Art. XI.

1. *Der Leberthran als Heilmittel, auf Grundlage vielfacher Thatsachen und Versuche an Thieren, vom physiologisch-pathologischen Standpunkte dargestellt.* Von Dr. Hermann Klencke.—*Leipzig*, 1842. 8vo, pp. 127.

*Cod Liver Oil, as a curative means, physiologico-pathologically illustrated by a variety of facts and experiments on animals.* By Dr. Hermann Klencke.—*Leipzig*, 1842.

2. *Beobachtungen über den Leberthran, besonders in seiner Anwendung gegen scrophulöse Krankheitsformen.* Von Eduard Adolph Panck, Dr. Medicinæ russisch-Kaiserlichem Hofrathe, &c. in Moskau.—*Hamburg*, 1842. 8vo, pp. 41.

*Observations on Cod Liver Oil, particularly in its action on Scrofulous Diseases.* By Dr. Edward Panck, of Moscow. [From Vol. XX. Part III. (July, 1842) of the Hamburg "Zeitschrift für die gesammte Medicin.]

In a former Number (XXV) of this Journal we reviewed a treatise, by Dr. Bennett, on the therapeutical uses of the cod-liver oil. We are induced to revert to this subject, from having before us the two works, the titles of which head this article.

In the course of Dr. Klencke's work very important questions on the theory of digestion, such as the manner and nature of chymification, chylification, the uses of the bile and of the pancreatic juice, are introduced, and handled with more or less amplitude. In fact, although an inquiry into the therapeutical merits of cod-liver oil is professedly the main subject, yet actually it proves to be rather an incidental and secondary one, in Dr. Klencke's treatise; the physiological disquisitions on digestion being the principal, both as regards the attention and the space allotted to them. As, however, the object of the present article is to place before the reader simply any additional evidence, which Dr. Klencke's work may contain, of the value of the oil as a remedial agent, rather than to embark in a speculative discussion on digestion, and collateral questions, we shall be compelled, in a great measure, to pass over, for the present at least, those portions of the volume which are devoted to these subjects.

Now, on referring to our former notice, and on again looking through the volume of Dr. Bennett, we find that both that gentleman and we have already pretty well fulfilled our respective tasks. In other words, we observe, that while Dr. Bennett's treatise embodies, to a considerable extent, the principal facts already ascertained in regard to the therapeutical uses of the cod-liver oil, we have on our part given a very fair summary of, and passed what we conceive to be a very unexceptionable judgment on the contents of Dr. Bennett's volume. Still, as Dr. Klencke's work forms a completer and abler exposition than that of Dr. Bennett—moreover, as it presents evidence more circumstantial and decisive of the success of the cod-liver oil treatment, we deem it worth while, as we have already said, to devote a short space to a further consideration of the alleged—we may now say established—antistrumous and other virtues of the oleum jecoris aselli. It is almost unnecessary to observe, that we shall refrain from repeating here any of the facts or information contained in our former article.

Dr. Ascherson conceives that oil-globules are essential to the formation of the elementary cells of animal tissue, and that these globules are constituted from fluid fat and albumen. He observed that on fat, in a fluid state and albumen being brought into contact, a small quantity of the latter instantly formed a case or pellicle round a globule of the former, and that from the globules thus produced, as we have already stated, the elementary cells of all tissues are formed. Now, according to Dr. Ascherson, while on the one hand normal bile furnishes a due supply of oil, chyme supplies a due proportion of albumen, and thus, by the union of these in the manner already described, the formative rudiments of animal tissue are produced.

Dr. Klencke, while admitting what Dr. Ascherson alleges as to the mutual effects of fluid fat and albumen, denies (p. 26) that the phenomenon is to be regarded as having any true resemblance to organic cellular formation, since the organic cell is first developed as a nucleus, and the periphery is formed posteriorly to the centre. He also observes that the oil and albumen of the globules could not enter the lacteals without being previously dissociated, if not decomposed; and he further notices that chyme, *prior* to its admixture with bile, contains fat. Throughout the whole of his work, Dr. Klencke assumes that fat used as food and reduced to albumen, may be reconverted to fat, in the mesenteric glands, in the blood, in the cells of the parenchyma of the liver, and in the gall-bladder; and that this reconversion is favoured by a state of quiescence, as, for example, that produced by sedentary habits.

The author seems to think that cod-liver oil owes its power of improving chylification to its resemblance to bile, to which he conceives the oil to be a succedaneum. (p. 35.) Bile and the cod liver oil resemble each other in so far as both contain fat, resin, and similar saline constituents. (p. 20.) He does not think that the therapeutical action of the oil is due to its containing iodine, or bromine, or resin; but that in its oleaginous nature, and its general character as an animal oil, consist its medicinal efficacy (pp. 18, 19, 39). Elsewhere it is asserted that an aliment of the fat of pork or bacon produces all the beneficial consequences of the cod-liver oil. Its physiological properties are more minutely defined as consisting in stimulating the lymphatic and capillary systems, and the functions of secretion and excretion, and in improving nutrition; the deterioration of which is one of the omniform "bad effects of scrophulosis." It also replenishes the blood with an energetic and rich plasma, and, amid the activity of the vital processes to which it gives rise, promotes the absorption of all scrofulous depositions. (p. 78.) According to Dr. Klencke, the use of the oil serves to develope in the chyle those non-nucleated corpuscles, in the blood those colourless globules, and in the lymph the molecules peculiar to it, which furnish the formative constituents of the tissues; and the rarity and plenty, respectively, of which corpuscles in the three fluids now named are a sign of normal or of cachectic assimilation. (p. 64.) In short, the principal therapeutical excellence of the oil seems to consist in its power of acting as a substitute for the bile, when that secretion is either vitiated in quality or deficient in quantity.

That cod-liver oil possesses the property now ascribed to it Dr. Klencke infers from a variety of experiments on animals, principally cats and dogs. He found that after ligature of the ductus communis, the chyle, in cases in which no cod-liver oil was given, wanted the non-nucleated corpuscles, and showed, on being allowed to rest, no fatty stratum; but exhibited both of these when the oil had been administered prior to the death and dissection of the animal. (pp. 64-70.)

There is a disease to which cats are subject closely resembling scrofula in man. The animal is scabby, emaciated; its belly is tumid; it has a ravenous appetite; its eyes are tearful, the lids inflamed, and the nose exhibits sores. In this state even the endermic application of the oil appears to operate beneficially. A cat in the condition described was plunged, up to the neck, twice in the day, into "ordinary train-oil," and then kept wrapped in a flannel soaked in the same. The effect seemed to be equal to that of taking the oil internally.

The author also recommends inhalation of "an atmosphere impregnated with the oil gas" in cases of tuberculosis. The case of a lady labouring under "full-formed [ausgebildeter] tuberculosis" and hectic fever, is recorded, in which a cure was effected in four months by this method of treatment; although the oil seems to have been employed in other forms besides that of inhalation. The author conceives that, used in the atmospheric form, the oil acts partly by endosmosis; partly by "its stimulating effects on the capillary system and peripheric nerves of the lungs." (p. 106.)

From the fact that the resin, which abounds in the darker-coloured sorts of the oil, appeared in the stools of one of the cats to which he administered it, Dr. Klencke infers that it is not to the resin that the efficacy of the oil is due; and he presumes from analogy that the resin of the bile, in like manner, is purely excrementitious, and exerts no positive influence in chylification.

In one respect the cod liver oil differs from bile, namely, in its reaction being acid, while that of bile is alkaline. (p. 25.) Yet we find that it is highly useful, and that too, probably, just on account of the reaction referred to, in cases of persons of biliary diathesis, the secretion of whose livers is very alkalescent and very inspissated. (p. 92.)

Towards the end of his work Dr. Klencke gives similar cautions and directions as to his cases proper for the exhibition of the oil, which Dr. Bennett has done in his treatise, and which we have quoted in our Twenty-fifth Number, page 201. Thither we refer the reader.

We are surprised by a statement which occurs at an early part of Dr. Klencke's work. He says that between the action of the three principal sorts of oil, there is as great a difference as between that of the various preparations of mercury. (p. 4.) We shall therefore briefly state the circumstances proper for the exhibition of these three sorts respectively; premising that, so far as regards colour, they are distinguishable into a tar-like black species, a fluid red, and a fluid brown and golden yellow species. (p. 3.)

In atrophic states, and states of cachectic assimilation, in which the indication is to improve chylification, the oleum subflavum, or the clearer and purer sort, is the suitable one. It is adapted for children and adults, with marked gastric irritability, but the action of whose bowels is languid. (p. 122.) But if, after cachectic symptoms, there manifests itself a tendency to morbid formations or depositions, and it seems desirable to correct the constitution of the fluids by a stimulant action on the nervous, vascular, and secretory systems, the dark oil, or that which abounds with resin, and even the fusco-empyreumatic species, is indicated. The author states that he has cured gouty and rheumatic cases more speedily by these last sorts than by the subflavous variety. These diseases, in adults, require the red oil, which species is also peculiarly adapted for cases of nervous irritation accompanied by dyscrasia, as well as "inflammatory affections which seem to result from a vitiated state of the plasma.". (p. 123.)

Dr. Klencke and his "colleagues" appear to have treated successfully, by cod-liver oil, five cases of tuberculosis; two cases of acute hydrocephalus in children; three rachitic cases; two cases of caries scrophulosa; one case of chronic gout, with a cachectic constitution; four cases of spinal irritation, accompanied now with paralysis, now with pain in the backbone and pericardium; one case of epilepsy; nineteen cases of chronic skindisease; one case of the "purulent diathesis, without marked gastritic symptoms;" eight cases of inflammatory affection of the glands in all stages and varieties; two cases of chlorosis; one case of fluor albus; one case of gastro-malacia. (p. 107.)

The author directs, as the smallest dose to begin with, three tablespoonfuls of the oil daily; which quantity may be increased daily to twenty, or until the patient comes to live almost entirely on the oil.

The second essay which heads this article forms a long original contribution of 40 pages, in the "Hamburg Zeitschrift für die gesammte Medicin." We notice it on account of the strong testimony which it yields in favour of Oleum Jecoris Aselli, as a remedial means in a variety of diseases, but chiefly in those of a strumous character. Although, however, a useful practical dissertation, it does not appear to contain anything very new, or calling for particular notice.

"I have been," says Dr. Panck, "eight years, physician to the Alexandrian Orphan house, in Moscow, which contains three hundred children, of both sexes, and various ages. Here I have had much to do with scrofulous affections, and I must confess that, in the first years of my practice, when I did not employ the oil, the result of my treatment was much less favorable than of later years, when I did employ it." (p. 2.)

After detailing, with considerable minuteness, fifteen cases of various kinds, some cured, others not, he concludes with the following inference:

"From these cases we see how beneficial the oil is, in various forms of the scrofulous dyscrasy. Obstructions and indurations in the lymphatic system, congestions and infarctus (anschoppungen) in the glandular organs of the abdomen, of the liver and spleen, affections of the mucous membranes, swellings of the salivary glands, serous effusions, diseases of the fibrous tunics and periosteum, &c.; all these frequently find, in the cod-liver oil, when fairly indicated, and properly administered, an indispensable therapeutic agent." (p. 40.)

In conclusion, we beg to state that we still retain the belief which we formerly expressed, that the cod-liver oil will not, in practice, realize all the expectations which Dr. Bennett's treatise was calculated to excite in its favour, and we would make the same remark of the treatises now under review. For example, we are firmly persuaded that by far the majority of cases of chronic gout and rheumatism, particularly in adults, will *not* own the Ol. Jecoris Aselli as a radical curative means. We also believe that, in a majority of cases, it will fail to eradicate the scrofulous diathesis, or to affect any lasting amendment in cases of tuberculous lungs, whether the tubercles be solid or soft. And we fear that the case of phthisis, referred to by Dr. Bennett, (Treatise, p. 133,) "in which a vomica, with perfect pectoriloquy, was detected under the right clavicle, and which, "by means of the oil," had "a successful result," is destined to have but very "few and far between" parallels indeed. Still, the strong and recurring evidence of the beneficial effects of the Oleum Jecoris Aselli (and we can add our own on a small scale) forbids us to doubt that it is an agent of considerable efficacy in some cases; and we trust that our present notice of it will induce members of the profession to embrace suitable opportunities of testing its merits, and of reporting the results of their observations.

